# Do acute coronary events affect lipid management and cholesterol goal attainment in Germany? Results from the Dyslipidemia International study II

**DOI:** 10.1007/s00508-018-1375-3

**Published:** 2018-09-03

**Authors:** Anselm K. Gitt, Johannes Rieber, Rainer Hambrecht, Johannes Brachmann, Kristof Graf, Martin Horack, Barbara Karmann, Ami Vyas, Dominik Lautsch, Baishali Ambegaonkar, Philippe Brudi

**Affiliations:** 1 Herzzentrum Ludwigshafen, Ludwigshafen, Germany; 2 Stiftung IHF—Institut für Herzinfarktforschung Ludwigshafen, Bremser Str. 79, 67117 Ludwigshafen, Germany; 3 Klinikum Bogenhausen, Munich, Germany; 4 Klinikum Links der Weser, Bremen, Germany; 5 Klinikum Coburg, Coburg, Germany; 6 Jüdisches Krankenhaus Berlin, Berlin, Germany; 7 MSD SHARP & DOHME GmbH, Haar, Germany; 8 School of Public Health, Department of Epidemiology, Rutgers University, Piscataway, NJ USA; 9 Merck & Co., Inc., Kenilworth, NJ USA; 10 College of Pharmacy, Department of Pharmacy Practice, University of Rhode Island, Kingston, RI USA

**Keywords:** Acute coronary syndrome, Myocardial infarction, Dyslipidemias, Cholesterol, LDL

## Abstract

**Objective:**

To document utilization of lipid-lowering therapy, attainment of low-density lipoprotein cholesterol target values, and cardiovascular outcomes in patients hospitalized for acute coronary syndrome in Germany.

**Methods:**

The Dyslipidemia International Study II was a multicenter, observational study of the prevalence of dyslipidemia and lipid target value attainment in patients surviving any acute coronary syndrome event. Among patients on lipid-lowering therapy for ≥3 months, use of lipid-lowering therapy and lipid profiles were assessed at admission and again at 120 ± 15 days after admission (the follow-up time point). Multivariate logistic regression was used to identify variables predictive of low-density lipoprotein cholesterol target value attainment in patients using lipid-lowering therapy.

**Results:**

A total of 461 patients hospitalized for acute coronary syndrome were identified, 270 (58.6%) of whom were on lipid-lowering therapy at admission. Among patients on lipid-lowering therapy, 90.7% and 85.9% were receiving statin monotherapy at admission and follow-up, respectively. Mean (SD) low-density lipoprotein cholesterol levels in patients on lipid-lowering therapy were 101 (40) mg/dl and 95 (30) mg/dl at admission and follow-up, respectively. In patients with data at both admission and follow-up (*n* = 61), low-density lipoprotein cholesterol target value attainment rates were the same (19.7%) at both time points. Smoking was associated with a 77% lower likelihood of attaining the low-density lipoprotein cholesterol target value.

**Conclusion:**

Hospitalization for an acute event does not greatly alter lipid management in acute coronary syndrome patients in Germany. Both lipid-lowering therapy doses and rates of low-density lipoprotein cholesterol target value attainment remained essentially the same several months after the event.

## Introduction

Acute coronary syndrome (ACS) is a serious and life-threatening clinical manifestation of atherosclerosis. It is characterized by a thromboembolic event leading to a sudden reduction in blood flow to the heart [1]. An ACS presents as one of several sub-types, including ST segment elevation myocardial infarction (STEMI), non-ST elevation myocardial infarction (NSTEMI), and unstable angina. European Society of Cardiology (ESC) guidelines for the management of ACS acknowledge that secondary prevention of cardiovascular events requires treatment of dyslipidemia, if present [1, 2]. Specifically, long-term treatment of low-density lipoprotein cholesterol (LDL-C) serum levels to a target value of <70 mg/dl (1.8 mmol/l) is recommended, with high-dose statins and ezetimibe as the preferred lipid-lowering therapy (LLT; [2, 3]). These targets have been confirmed by the guidelines for the management of dyslipidemia published by the European Society of Cardiology (ESC) and the European Atherosclerosis Society (EAS) task force in 2011 [4].

Germany has made several improvements in the management of cardiovascular disease risk factors in ACS patients. Rates of treatment of dyslipidemia in patients with a history of ACS increased from 35% to 87% between 1995 and 2007 [5], and nationwide implementation of smoking restrictions in 2007–2008 was followed by 13.3% and 8.1% declines, respectively, in the annual rates of hospitalization for angina pectoris and myocardial infarction (MI; [6]); however, rates of hospitalization for MI in Germany remain well above the median for European countries [7]. An analysis of the German 2L registry of patients with coronary heart disease (CHD) showed that most patients treated for dyslipidemia received low-intensity statin regimens [8]. Not surprisingly, the Dyslipidemia International Study (DYSIS) found that, among statin-treated patients in Germany, 58.1% failed to attain the LDL-C target value for high risk patients (<100 mg/dl; 2.6 mmol/l) [9], and a large German population-based cross-sectional study found that among statin-treated CHD patients, the estimated 10-year risk of a coronary event was 35.1%, well above the threshold for LLT [10].

Given the state of cardiovascular management in Germany and the current guidelines for treatment of ACS, the primary objective of the second DYSIS (DYSIS II) was to document utilization of LLT, LDL-C target value attainment, and cardiovascular health outcomes in patients hospitalized for ACS in Germany.

## Patients, materials and methods

### Study design

The DYSIS II is an international, multicenter, observational study being conducted throughout Europe, Asia, and the Middle East to determine the prevalence of dyslipidemias and lipid target value attainment in patients with stable CHD and in patients hospitalized for an ACS event. The ACS cohorts were assessed longitudinally as described later. All data were collected via a web-based data collection form using software developed by the Institute for Heart Infarction Research (*Institut für Herzinfarktforschung*, IHF) in Ludwigshafen, Germany.

In the German ACS cohort, 21 sites participated in data collection from consecutive patients. Acute care centers were selected to be representative of the acute and ambulatory treatment of secondary prevention in Germany. The patient recruitment period was May 2013 to July 2014. Data were collected by clinical examination and from medical charts at admission to the hospital, and again via a telephone interview at 120 ± 15 days after admission (the follow-up time point). At the time of enrolment, patients were given a booklet and instructed to take it with them when they next visited their physician post-discharge (from current hospitalization). The booklet was filled out during the follow-up office visit by the physician and registered the patient’s lipid profile and other basic patient characteristics. The information in the booklet was to be used by the patient during the follow-up telephone interview. The protocol was approved by local and regional institutional review boards as per local regulations.

### Study sample

Patients included in the current analysis were ≥18 years of age, had been hospitalized for ACS in Germany, and had a full lipid profile based on blood drawn within 24 h of admission. Patients must have been on LLT for ≥3 months, or not taking LLT at all, at the time of admission to the hospital. Patients taking LLT for <3 months were excluded from the analysis. Each patient provided written informed consent specifying non-participation in any randomized clinical trials and would not do so for the duration of the study.

### Study definitions and outcome variables

In this study, ACS was defined as one or more of the following events: STEMI/left bundle branch block (LBBB), NSTEMI, or unstable angina. Demographic and clinical characteristics collected at admission included age, gender, body mass index, sedentary life style, smoking status, and family history of CHD. Comorbidities (e. g., hypertension, type 2 diabetes mellitus) and cardiovascular history (of CHD, MI, chronic renal failure, chronic kidney disease, stroke, or peripheral vascular disease) were also recorded. Obesity was defined according to the World Health Organization (WHO) criteria as having a body mass index >30 kg/m^2^. Hypertension was defined as current antihypertensive treatment, a previous diagnosis, or having blood pressure >140/90 mm Hg. Similarly, diabetes was defined as current treatment for diabetes, a previous diagnosis of diabetes, or a fasting plasma glucose level of ≥126 mg/dl. A sedentary life style was defined as <20–30 min of walking on <3–4 days per week. Stroke was either ischemic or hemorrhagic. Use of selected classes of cardiovascular medications (e. g., beta-blockers, calcium channel blockers, diuretics, angiotensin-converting enzyme (ACE) inhibitors, antiplatelet agents) and laboratory values of hemoglobin A1c and serum glucose were also recorded at admission.

Patients were divided into two subgroups, treated or untreated, based on their lipid treatment status at admission, as defined above. Use of LLTs at the time of the lipid test was determined by chart review at admission and by patient report at follow-up. The following mutually exclusive classes of LLT were assessed: statin monotherapy, non-statin monotherapy, statin plus ezetimibe, and statin plus other non-statin therapy (“other” non-statins included fibrates and omega-3 fatty acids). The statins assessed were simvastatin, atorvastatin, rosuvastatin, pravastatin, lovastatin, and fluvastatin. Atorvastatin and simvastatin dose equivalents were calculated based on clinical trial data on the LDL-C-lowering efficacy of various statins [11]. Attainment of lipid target values was assessed among treated patients at admission and follow-up, using lipid values determined within 24 h of admission and lipid values determined between admission and the follow-up interview, respectively. The lipid profile included measurement or calculation of serum levels of total cholesterol, LDL-C, high-density lipoprotein cholesterol (HDL-C), triglycerides, and non-HDL-C. The LDL-C target values were assigned according to the patient’s cardiovascular risk, which was determined using two methods. First, pre-ACS risk status (i.e. very high, high, moderate or low) was determined for all patients based on selected patient characteristics, and second, all patients were classified as being at very high risk because of the ACS event leading to hospitalization. Target values for LDL-C for very high risk, high risk, moderate risk, and low risk patients were defined according to the 2011 ESC/EAS guidelines as <70 mg/dl, <100 mg/dl, <115 mg/dl, and <130 mg/dl, respectively [4]. Per the same guidelines, the non-HDL-C target value was <100 mg/dl [4]. The median distance to the LDL-C target value was calculated for patients who had not attained the LDL-C target value on the date of the lipid profile. Attainment of the secondary non-HDL-C target value (<100 mg/dl) was also assessed at admission and follow-up. Cardiovascular health outcomes assessed at follow-up were rehospitalization, MI, stroke, percutaneous coronary intervention, and coronary artery bypass graft.

### Statistical analysis

This study analyzed the ACS patients on LLT at admission through the follow-up time point. Unless otherwise specified, the designations of “treated” or “on LLT” refer to the treatment status at admission, regardless of the treatment status at follow-up. Demographic and clinical characteristics, use of cardiovascular medications, and cardiovascular outcomes were compared between patients on LLT and not on LLT at admission using χ^2^ or Mann-Whitney-Wilcoxon tests. Lipid profiles, LDL-C target value attainment, and types of LLT used at admission and follow-up were assessed descriptively in patients on LLT. In all univariate analyses, continuous variables are presented as means and standard deviations (SDs) or medians and interquartile ranges (IQRs), and categorical variables are presented as numbers and/or percentages. Multivariate logistic regression was used to identify variables predictive of LDL-C target value attainment in patients on LLT at admission. Covariates, including age, gender, obesity, current smoking, sedentary life style, stable angina, chronic kidney disease, type 2 diabetes mellitus, history of congestive heart failure, hypertension and statin dose (i. e., atorvastatin dose equivalent), were chosen based on their potential to affect LDL-C target value attainment, and no statistical selection methods were applied. Assessment of cardiovascular outcomes at follow-up was done using Kaplan-Meier analysis, with *P* values calculated by a log-rank test. SAS version 9.3 (Cary, NC, USA) was used for all calculations. In all analyses, a *P* value <0.05 was considered statistically significant.

## Results

### Characteristics of the study population

**Fig. 1:**
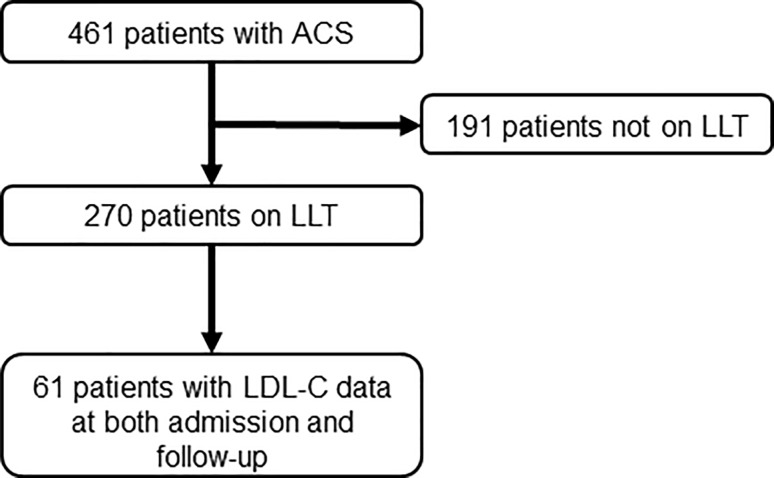
Flowchart for patients in the study. *ACS* acute coronary syndrome, *LLT* lipid-lowering therapy, *LDL-C* low-density lipoprotein cholesterol

The study identified 461 patients hospitalized for ACS in Germany in 2013–2014 (Fig. 1). The mean (SD) age of the study population was 64.1 (11.7) years, and 75.5% were male (Table 1). More than half of the patients had hypertension (80.9%) or documented CHD (56.9%). The most frequently used types of cardiovascular medication were beta blockers (63.1%), acetylsalicylic acid (57.8%), and ACE inhibitors (46.4%). Mean (SD) values of hemoglobin A1c and serum glucose were 6.4% (1.5%) and 131.8 (46.4) mg/dl, respectively. During the hospital stay, 388 patients (84.2%) underwent echocardiography, 310 (67.2%) received a percutaneous coronary intervention, and 194 (42.1%) underwent coronary angiography (data not shown).

**Table 1:** Demographic and clinical characteristics of the study population^a^

	All patients (*N* = 461)	Patients on LLT (*N* = 270)	Patients not on LLT (*N* = 191)	*P* value^b^
*Age, mean (SD) years*	64.1 (11.7)	66.4 (10.5)	60.8 (12.6)	<0.001
*Male*	348 (75.5)	212 (78.5)	136 (71.2)	0.07
*Obese* ^*c*^	149 (32.4)	100 (37.2)	49 (25.7)	<0.01
*Hypertension*	373 (80.9)	244 (90.4)	129 (67.5)	<0.001
*Type 2 diabetes mellitus* ^*c*^	133 (29.0)	102 (37.9)	31 (16.3)	<0.001
*Oral medication for control of diabetes*	72 (15.6)	57 (21.1)	15 (7.9)	0.54
*Sedentary life style* ^*c*^	94 (24.5)	49 (21.6)	45 (28.7)	0.11
*Current smoker*	126 (27.3)	58 (21.5)	68 (35.6)	<0.001
*Documented CHD* ^*c*^	256 (56.9)	214 (80.8)	42 (22.7)	<0.001
*History of MI* ^*c*^	133 (30.1)	122 (47.7)	11 (5.9)	<0.001
*History of CRF/CKD*	54 (11.7)	44 (16.3)	10 (5.2)	<0.001
*Family history of CHD* ^*c*^	191 (48.0)	124 (54.9)	67 (39.0)	<0.01
*History of stroke (ischemic or hemorrhagic)* ^*c*^	26 (5.9)	21 (8.1)	5 (2.7)	<0.05
*History of peripheral vascular disease* ^*c*^	39 (8.6)	32 (12.0)	7 (3.7)	<0.01
*Type of ACS at admission*				
STEMI/LBBB MI	142 (30.8)	54 (20.0)	88 (46.1)	<0.001
NSTEMI	187 (40.6)	119 (44.1)	68 (35.6)	0.07
Unstable angina	132 (28.6)	97 (35.9)	35 (18.3)	<0.001
*Medication use* ^*c*^				
Beta-blockers	286 (63.1)	220 (81.8)	66 (35.9)	<0.001
Calcium channel blockers	79 (17.6)	60 (22.5)	19 (10.4)	<0.001
Diuretics	121 (26.7)	93 (34.6)	28 (15.2)	<0.001
ACE inhibitors	210 (46.4)	162 (60.2)	48 (26.1)	<0.001
Angiotensin receptor blockers	77 (17.1)	51 (19.2)	26 (14.1)	0.16
Acetylsalicylic acid	262 (57.8)	212 (78.5)	50 (27.3)	<0.001
Other antiplatelets	90 (19.5)	73 (27.0)	17 (8.9)	<0.001
Clopidogrel	49 (10.6)	38 (14.1)	11 (5.8)	0.35
Prasugrel	16 (3.5)	15 (5.6)	1 (0.5)	0.15
Ticagrelor	23 (5.0)	18 (6.7)	5 (2.6)	0.69
Other	2 (0.4)	2 (0.7)	0 (0.0)	0.49
*Laboratory values*				
Hemoglobin A1c, mean (SD) %	6.4 ± 1.5	6.7 ± 1.4	6.1 ± 1.6	<0.01
Serum glucose, mean (SD) mg/dl	131.8 ± 46.4	133.6 ± 50.2	129.5 ± 40.9	0.96

*ACS* acute coronary syndrome, *ACE* angiotensin-converting enzyme, *CHD* coronary heart disease, *CKD* chronic kidney disease, *CRF* chronic renal failure, *LBBB* left bundle branch block, *LLT* lipid-lowering therapy, *MI* myocardial infarction, *NSTEMI* non-ST elevation myocardial infarction, *SD* standard deviation, *STEMI* ST segment elevation myocardial infarction

^a^Data are presented as numbers and percentages unless otherwise indicated

^b^*P* values reflect χ^2^ or Mann-Whitney-Wilcoxon tests between values for treated and untreated patients

^c^Values were calculated based on the number of patients with available data rather than the total study or subgroup population

A total of 270 patients (58.6%) were on LLT at admission and 191 were not (Fig. 1; Table 1). Patients not on LLT at hospital admission were younger compared to those on LLT (60.8 versus 66.4 years, *P* < 0.001) and had significantly lower rates of obesity (25.7% versus 37.2%, *P* < 0.01), hypertension (67.5% versus 90.4%, *P* < 0.001), diabetes (16.3% versus 37.9%, *P* < 0.001), documented CHD (22.7% versus 80.8%, *P* < 0.001), and history of MI (5.9% versus 47.7%, *P* < 0.001). The use of almost all types of cardiovascular medications was significantly less frequent in patients not on LLT than those on LLT (Table 1), and patients not on LLT were more frequently current smokers (35.6% versus 21.5%, *P* < 0.001) and more often presented with STEMI/LBBB MI (46.1% versus 20.0%, *P* < 0.001).

### Use of lipid-lowering therapies

Among the 270 treated patients, 97.8% were receiving statins at admission (Table 2), and the most commonly used statins were simvastatin (69.3%) and atorvastatin (21.9%; Table 2). In addition, 8.1% of patients were taking non-statin LLT. At admission, the mean (SD) atorvastatin dose equivalent was 18 (12) mg/day (Table 3). At the time of the follow-up interview, only 86.4% of patients on LLT at admission reported taking statins (Table 2). Simvastatin use decreased to 55.4% at follow-up, and atorvastatin held steady at 21.5% (Table 2). The mean (SD) atorvastatin dose was 22 (15) mg/day (Table 3).

**Table 2:** Lipid-lowering therapy at discharge and follow-up

	Discharge (*N* = 270)	Follow-up^a^ (*N* = 242)
**Statins, ** ***n*** ** (%)**	264 (97.8)	209 (86.4)
*Atorvastatin, n (%)*	59 (21.9)	52 (21.5)
Mean (SD) dose, mg/day	37 ± 14	38 ± 16
*Fluvastatin, n (%)*	8 (3.0)	7 (2.9)
Mean (SD) dose, mg/day	48 ± 21	51 ± 20
*Lovastatin, n (%)*	0 (0.0)	0 (0.0)
Mean (SD) dose, mg/day	–	–
*Pravastatin, n (%)*	10 (3.7)	12 (5.0)
Mean (SD) dose, mg/day	32 ± 13	31 ± 12
*Rosuvastatin, n (%)*	0 (0.0)	0
Mean (SD) dose, mg/day	–	–
*Simvastatin, n (%)*	187 (69.3)	134 (55.4)
Mean (SD) dose, mg/day	34 ± 12	33 ± 12
*Pitavastatin, n (%)*	0 (0.0)	0
Mean (SD) dose, mg/day	–	–
**Non-statins, ** ***n*** ** (%)**	22 (8.1)	22 (9.1)
*Ezetimibe, n (%)*	19 (7.0)	18 (7.4)
*Fibrates, n (%)*	2 (0.7)	3 (1.2)
*Nicotinic acids, n (%)*	0 (0.0)	0 (0.0)
*Laropiprant, n (%)*	0 (0.0)	0 (0.0)
*Omega-3 fatty acids, n (%)*	1 (0.4)	0 (0.0)

^a^Although 242 patients were included in the follow-up analyses, only 65, 61, 62, 56, and 57 patients had lipid profile data for total cholesterol, LDL-C, HDL-C, triglycerides, and non-HDL-C, respectively, at follow-up

**Table 3:** Lipid profiles, distance to target, and dose equivalents among treated patients at admission and follow-up

	Admission (*N* = 270)	Follow-up^a^ (*N* = 242)
*Lipid concentrations, mg/dl*		
Total cholesterol, mean (SD)	174 (50)	165 (32)
LDL-C, mean (SD)	101 (40)	95 (30)
HDL-C, median (IQR)	43 (36, 50)	50 (40, 59)
Triglycerides, median (IQR)	122 (85, 185)	128 (95, 164)
Non-HDL-C, median (IQR)	121 (98, 152)	115 (98, 143)
*Distance to LDL-C <70* *mg/dl, median (IQR)*^*b*^	34 (17, 60)	31 (11, 55)
*Atorvastatin dose equivalent, mean* *±* *SD mg/day*^*c*^	18 ± 12	22 ± 15

*HDL-C* high-density lipoprotein cholesterol, *IQR* interquartile range, *LDL-C* low-density lipoprotein cholesterol, *SD* standard deviation

^a^Although 242 patients were included in the follow-up analyses, only 65, 61, 62, 56, and 57 patients had lipid profile data for total cholesterol, LDL-C, HDL-C, triglycerides, and non-HDL-C, respectively, at follow-up

^b^Among patients not yet attaining the target level

^c^For comparison, the simvastatin dose equivalents were 37 ± 23 mg/day at admission and 44 ± 31 mg/day at follow-up

### Lipid profiles and lipid target value attainment

At admission, mean (SD) total cholesterol and LDL-C levels in patients on LLT were 174 (50) mg/dl and 101 (40) mg/dl, respectively (Table 3). Median (IQR) HDL-C, triglyceride, and non-HDL-C levels were 43 (36–50) mg/dl, 122 (85–185) mg/dl, and 121 (98–152) mg/dl, respectively. Among the 242 patients with follow-up data, values for total cholesterol, LDL-C, and non-HDL-C decreased at follow-up, while the levels of HDL-C and triglycerides increased (Table 3).

**Fig. 2:**
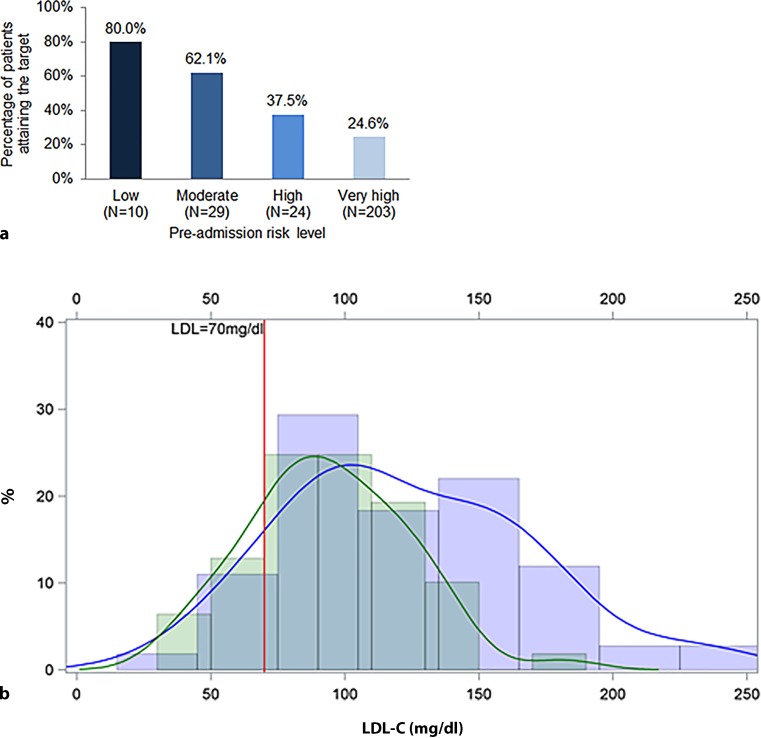
LDL-C target value attainment, by **a** pre-ACS risk level and **b** time point. **a** Risk levels were determined using chart data from the pre-ACS period. LDL-C targets were as follows: very high risk, <70 mg/dl; high risk, <100 mg/dl; moderate risk, <115 mg/dl; and low risk, <130 mg/dl. **b** Goal attainment is shown in a kernel density plot for the subgroup of 61 treated patients for whom LDL-C data were available at both admission (*blue line*) and follow-up (*green line*). The X‑axis shows LDL-C levels and the Y‑axis shows the percentage of patients. Follow-up data were collected 120 days after admission to the hospital. *LDL-C* low-density lipoprotein cholesterol

At admission, 27.8% of treated patients had attained the non-HDL-C target, and 31.6% had done so by the follow-up assessment (data not shown). Among treated patients with very high pre-ACS risk (*N* = 203), only 24.6% attained LDL-C target values at admission (Fig. 2a). The LDL-C target value attainment rates for patients classified pre-ACS as high, moderate, and low risk were 37.5%, 62.1%, and 80.0%, respectively (Fig. 2a).

When all patients on LLT (*N* = 270) were classified as very high risk because of their hospitalization for ACS, 21.9% had attained the LDL-C target value at admission (data not shown). Among those not attaining the LDL-C target, the median (IQR) distance to the target value at admission was 34 (17–60) mg/dl (Table 3). At the time of follow-up, 61 treated patients had data for LDL-C at both admission and follow-up, and 19.7% of them had attained the LDL-C target value (Fig. 2b). Among these 61 patients, target value attainment at admission was 19.7% (Fig. 2b). Among treated patients not attaining the LDL-C target value at follow-up, the median (IQR) distance to the target was 31 (11–55) mg/dl (Table 3).

In multivariate regression analyses of LDL-C target value attainment (Table 4), chronic kidney disease was associated with 3‑fold higher odds of attainment (odds ratio OR 3.12, 95% CI 1.29–7.55), while current smoking was associated with a 77% lower likelihood of attaining the LDL-C target value (OR 0.23, 95% CI 0.07–0.72).

**Table 4:** Predictors of LDL-C target value attainment in patients on LLT at admission^a^

	Odds ratio	95% CI	*P-*value
Age ≥70 years	0.71	0.33–1.52	0.376
Female	1.39	0.59–3.32	0.454
BMI >30 kg/m^2^	0.98	0.44–2.17	0.960
Current smoking	**0.23**	**0.07–0.72**	**0.012**
Sedentary life style	0.70	0.29–1.69	0.426
Chronic kidney disease	**3.12**	**1.29–7.55**	**0.012**
Type 2 diabetes mellitus	1.18	0.54–2.59	0.680
History of congestive heart failure	1.16	0.34–3.96	0.819
Hypertension	0.51	0.17–1.55	0.235
Statin dose (atorvastatin dose equivalent, mg/day)	1.02	0.988–1.05	0.220

*BMI* body mass index, *CI* confidence interval

^a^**Bold **print indicates statistical significance

## Discussion

The DYSIS II assessed lipid profiles and LDL-C target value attainment longitudinally in patients receiving LLT who were hospitalized for ACS in Germany. Approximately 20% of treated German ACS patients with data at both admission and follow-up attained the recommended LDL-C level of <70 mg/dl, with no apparent gains between hospitalization and follow-up. Over the same time interval, triglyceride levels actually increased. These findings suggest potential reasons for such poor outcomes: LLT at admission consisted mainly of statin monotherapy (primarily simvastatin), and the mean atorvastatin dose equivalent increased only slightly, from 18 to 22 mg/day, between admission and follow-up.

According to the 2011 ESC/EAS guidelines [4] and the more recently published 2016 ESC/EAS guidelines [12], all patients experiencing an ACS event should receive pharmacologic treatment. The EUROASPIRE surveys I–III showed that between 1995 and 2007 rates of treatment for lipid abnormalities among German patients with coronary events increased from 35.0% to 87.0% (*P* < 0.001) [5]. Other studies of German patients hospitalized for an ACS event (STEMI, NSTEMI, or unstable angina) reported statin treatment rates at discharge ranging from 73% to 94.6% [13–16]; however, EUROASPIRE IV found that in Germany low/moderate-intensity LLTs are much more frequently used than high-intensity LLTs [17], consistent with our finding that simvastatin was the most commonly used LLT in DYSIS II. Other studies have found that simvastatin is the most commonly prescribed statin in Germany [8–10] and that non-statins are used infrequently for lipid control [8, 9]. The 2016 ESC/EAS guidelines state that lipid values should be re-evaluated 4–6 weeks after ACS to determine whether lipid goals have been reached and if the therapeutic regimen needs to be adapted [12]. In this study only a slight increase in dose strength was noted, from 18 to 22 mg/day, between admission and follow-up (120 ± 15 days after admission). Very often statin treatment initiated during the hospital stay is done so with low dosages and is expected to be up-titrated during follow-up. Our data indicate that this up-titration is not taking place. Use of less effective LLTs and failure to increase the dose potency after a coronary event may explain the low attainment of the LDL-C target value in German ACS patients.

As has been elucidated in the past [8], office-based physicians in Germany will likely not intensify LLT in most cases after discharge from hospital. It is therefore proposed to discharge patients post-ACS with high intensity LLT, including a potent statin and ezetimibe. The newly available PCSK9 inhibitors are an additional option to treat ACS patients, but the use of this therapy is limited in Germany. Before using a PCSK9 inhibitor, all other available therapeutic options must be exhausted, e. g., use of a potent statin (e. g., 40 mg atorvastatin) and the combination with ezetimibe. Consistent use of this strategy would bring most patients to the treatment goal. Additionally, clear advice in the discharge letter concerning the treatment goal and the therapeutic options could help general practitioners in the clinical setting to optimize the patients’ therapy. The ACS patients in Germany should also be encouraged to take part in rehabilitation programs after an acute event to improve the individual prognosis. In terms of identifying those patients at highest risk, the validated TRS2P score provides a modern and easy to use tool [18].

Among patients with data at both admission and follow-up, the LDL-C target value attainment rate in the German ACS cohort of DYSIS II did not change from hospital admission to follow-up. In part because previous studies of lipid target value attainment in Germany have used different targets, the reported rates of attainment are somewhat higher than in DYSIS II. The DYSIS applied a target of <100 mg/dl to high-risk statin-treated patients, and accordingly 41.9% of high-risk patients, as well as 47.3% of patients with cardiovascular disease, attained the target value [9]. Other studies applying the <100 mg/dl target to statin-treated patients in Germany have found LDL-C target value attainment rates of ~43–50% [8, 19]. Similarly, among German patients undergoing inpatient cardiac rehabilitation after hospitalization for an acute coronary event, 69.6% attained an LDL-C level <100 mg/dl by the discharge date [14]. To our knowledge, DYSIS II is the first study to assess attainment of the more stringent <70 mg/dl target value in ACS patients in Germany.

The only variable positively associated with LDL-C target value attainment in the current study was chronic kidney disease, which was associated with 3‑fold higher odds of attainment. According to the 2016 ESC/EAS guidelines, patients with chronic kidney disease are considered as high-risk or very high-risk patients and should be treated accordingly [12]. Because these patients are treated as high or very high risk, regardless of LDL-C values, it is possible that the more aggressive treatment protocol resulted in higher odds of LDL-C attainment. Smoking, on the other hand, was associated with a 77% lower likelihood of attaining the LDL-C target value. This result is consistent with the results from the German cohort of DYSIS, which found smoking to be associated with higher odds of LDL-C non-attainment in statin-treated patients [9].

One limitation of this study is that adherence to treatment was not directly assessed. Although most patients reported receiving a statin prescription at follow-up, actual medication-taking behavior was not queried. Previous studies in Germany suggest that adherence to statins falls by 20–35% within the first year of use [13, 19, 20]. Secondly, a follow-up of 120 ± 15 days was too short to observe any changes in therapy or cardiovascular outcomes. A long-term follow-up of the patients would be of interest to evaluate any changes of therapy in the longer term of the disease. Per the findings of de Lemos et al. [21], the effect of statin treatment on cardiovascular event risk reduction may not be evident until more time has passed. Finally, data collection by telephone at follow-up may not have been as accurate as the medical chart review used for data collection at admission and may have affected the direction and degree of changes observed over time.

In conclusion, this study of the German ACS cohort of DYSIS II showed that LDL-C target value attainment is suboptimal among very high-risk patients on LLT. Hospitalization for an ACS event did not greatly alter lipid management in ACS patients. Both LLT doses and rates of LDL-C target value attainment remained essentially the same several months after the event. These results indicate that LLTs are not utilized in an efficient manner in ACS patients in Germany, allowing cardiovascular mortality to remain high. An LLT should be administered according to the latest guidelines, i. e., with higher doses and combination therapies, in order to help patients attain their LDL-C target value and reduce their risk of cardiovascular events.
